# Prevalence of dyslipidemia and its association with other coronary artery disease risk factors among urban population in Southeast of Iran: results of the Kerman coronary artery disease risk factors study (KERCADRS)

**DOI:** 10.1186/s40200-016-0268-0

**Published:** 2016-10-21

**Authors:** Hamid Najafipour, Mostafa Shokoohi, Gholamreza Yousefzadeh, Behzad Sarvar Azimzadeh, Gholamreza Moshtaghi Kashanian, Mohamad Mehdi Bagheri, Ali Mirzazadeh

**Affiliations:** 1 Physiology Research Center, Institute of Neuropharmacology, Kerman University of Medical Sciences, Kerman, Iran; 2 Research Center for Modeling in Health, Institute for Futures Studies in Health, Kerman University of Medical Sciences, Kerman, Iran; 3 Endocrinology and Metabolism Research Center, Institute of Basic and Clinical Physiology Sciences, Kerman, Iran; 4 Department of Internal Medicine, Kerman University of Medical Sciences, Kerman, Iran; 5 Cardiovascular Research Center and Department of Cardiology, Kerman University of Medical Sciences, Kerman, Iran; 6 Global Health Sciences, University of California, San Francisco, CA USA

**Keywords:** Dyslipidemia, Prevalence, Coronary artery disease, Prediction, KERCADRS

## Abstract

**Background:**

Despite the importance of identifying and screening dyslipidemia to prevent coronary artery diseases CAD(Coronary Artery Disease), little information is available on dyslipidemia in our large area. So the present study aimed to assess the management status of lipid abnormalities and its association with other CAD risk factors in an urban population of southeast of Iran.

**Methods:**

This cross-sectional study was a part of the Kerman coronary artery disease risk factor study KERCADRS (Kerman coronary artery disease risk study) as a population-based, epidemiological research among 5900 individuals aged 15 to 75 years who were residents of Kerman city, the largest city in Southeast of Iran. Lipid profile was assessed using enzymatic laboratory methods.

**Results:**

In total, 5558 persons from 5899 participants were assessed in whom 45.1 % were male and 54.9 % female. Overall 20.9 % had borderline level of cholesterol (200–239 mg/dl) and 8.7 % suffered from hypercholesterolemia (≥240 mg/dl). The prevalence of undiagnosed dyslipidemia (UDL) was 16.8 % and of diagnosed dyslipidemia (DDL) was 13.2 % that both UDL and DDL were more prevalent in women. Also, UDL was more revealed in third and fourth age decades. Advanced age, anxiety, obesity (BMI ≥30 Kg/m^2^), and family history of dyslipidemia predicted dyslipidemia in study population.

**Conclusion:**

The overall prevalence of UDL was higher than of DDL, and was significantly influenced by advanced age, anxiety, obesity, and family history of dyslipidemia. The data showed that our health care management system should improve its strategies to reduce the burden of this important CAD risk factor.

## Background

Over the recent decades, many researches have consistently indicated the increased burden of lipid profile abnormalities and its high mortality, morbidity, and medical costs [1]. Dyslipidemia has been clearly identified as an important major risk factor for cardiovascular diseases that are the first cause of death in the developed and developing countries [2]. The World Health Organization recently reported that dyslipidemia is significantly associated with more than half of global cases of ischemic heart disease [3]. In fact, dyslipidemia is not a single primary phenomenon, but is a broad term that refers to inappropriate diet and lifestyle, as well as some genetic susceptibilities [2]. The literature on the epidemiology and economics of dyslipidemia is extensive. Quite literally, it has been demonstrated that a notable number of individuals have total cholesterol levels in excess of 200 mg/dL, and majority of affected ones have levels greater than 240 mg/dL, which is considered a high level necessitating treatment [4–6]. Despite the importance of identifying and screening dyslipidemia in every nation to prevent occurrence and progression of cardiovascular diseases, there are no proper and correct statistics on the incidence of dyslipidemia and its determinants in majority of nations [7, 8]. In this regard, awareness of the incidence and control state of dyslipidemia among high risk groups is now being increased, leading proper control of this serious risk factor in some areas [9]. Moreover, the recent report of the NCEP (National cholestrol education program) suggested that the primary approach to cholesterol lowering is making lifestyle changes in combination with medication to control their dyslipidemia [10]. The lack of enough knowledge to the status of dyslipidemia leads to inappropriately programming and treatment approaches.

Unfortunately, there is little information with respect to present status of lipid abnormalities and its association with other CAD risk factors especially mental health status among some Iranian population. This will lead to difficulties in scheduling suitable national managerial approaches for lipid lowering. Thus, the present study aimed to assess prevalence of lipid abnormalities and its associated CAD risk factors, and the effectiveness of health care system in management of this CAD risk factor in a relatively large population that is representative of the urban population in the Southeast of Iran.

## Methods

This cross-sectional study was a part of The Kerman coronary artery disease risk study (KERCADRS), as a population-based, epidemiological research among 5900 individuals aged 15 to 75 years. These are residents of Kerman city that addressed the epidemiological data regarding various coronary artery disease risk factors [11]. The study protocol was approved by the Research Ethics Committee at Kerman University of Medical Sciences, Kerman, Iran (Permission No. 88/110KA). Baseline demographic variables were collected either from the medical records or by interview -completed questionnaires at trial entry and comprised smoking status and opium use. Daily physical activities at home and workplace were recorded using WHO Global Physical Activity Questionnaire (GPAQ) [12]. To evaluate the intensity of physical activity, metabolic equivalent (MET) was used. MET is the use of energy in an adult individual while he/she is sitting. Moderate physical activity is considered as consuming energy four times, and high physical activity eight times in proportion to sitting. The questionnaire regarding risk profiles was completed by trained and certified medical staff. Beck Anxiety Inventory (BAI) and Beck Depression Inventory (BDI) [13] were of the questionnaires completed by the interviewers. Participants also underwent a clinical examination that included measurement of height, weight according to standardized protocols. Participants were categorized as overweight and obese if their body mass index were 25–29.9 and above 30 Kg/m^2^ respectively. Furthermore, blood samples were taken after at least 12 h of overnight fasting and FPG (Fasting Plasma Glucose)HbA1C (Hemoglobin A1C), total and HDL(High-Density Lipoprotein) cholesterol were measured. All lipid and lipoprotein measurements were made at a central laboratory. Total cholesterol was measured enzymatically with standard methods and triglyceride was measured via standard spectrophotometric technique. After the precipitation of low) particle with phosphotungstic acid, high-density lipoprotein (HDL) cholesterol was measured enzymatically in the supernatant by a modification of the method for total cholesterol. LDL(Low-Density Llipoprotein) cholesterol was calculated using the Friedewald formula (LDL = Total cholesterol – (HDL + TG (Serum Triglyceride)/5). Lipid profile was categorized based on the current measurements, history of diagnosis and taking drugs. The cut off points for these lipid profiles can be seen in Table 1. More information about the methodology of study has been published elsewhere [11].

**Table 1:** Lipid profiles categories based on the current measurements, history of diagnosis and taking drugs

Lipid profile categories	Average of at the time of recruitment (TC)	Average of at the time of recruitment (HDL-C)	Average of at the time of recruitment (LDL-C)	Average of at the time of recruitment (TG)	Self-reported of previously diagnosed	Self-reported of taking any anti- drug
Optimal/near-optimal serum concentration	<200	≥60 (negative risk factor)	<100 optimal (100–129 near-optimal)	<150	Negative	Negative
Borderline serum concentration	200–239	40–59 (men)	130–159	150–199	Negative	Negative
		50–59 (women)				
Undiagnosed	≥240	<40 men	160–189 high	200–499 high	Negative	Negative
High-risk/very high-risk serum concentration		<50 women	≥190 very high	≥500 very high		
Controlled - Diagnosed					Positive	Positive or Negative
Uncontrolled - Diagnosed					Positive	Positive or Negative

*TC* total cholesterol, *HDL-C* high density cholesterol, *LDL-C* low density cholesterol, *TG* triglyceride

### Statistical analysis

Results were presented as mean ± standard deviation (SD) for quantitative variables and were summarized by absolute frequencies and percentages for categorical variables. Continuous variables were compared using *t* test or ANOVA test or non-parametric Mann–Whitney U or Kruskal-Wallis tests whenever the data did not have normal distribution or when the assumption of equal variances was violated across the groups. Categorical variables were, on the other hand, compared using chi-square test or Fisher’s exact test when more than 20 % of cells with expected count of less than 5 were observed. Main predictors of dyslipidemia were determined by ANCOVA test adjusting baseline variables. For age-sex direct standardizations, we used Kerman population reported in census 2006. All prevalence rates were weighted according to the sampling weight (reciprocal of the probability of selection) and individual response rate. The statistical software SPSS version 20.0 for windows (SPSS Inc., Chicago, IL) was used for statistical analysis. *P* values of 0.05 or less were considered statistically significant.

## Results

Total population were 5899 persons from that, data of 5558 individuals were analyzed because their laboratory lipid measurement data was complete. From this population 45.1 % were male and 54.9 % were female, 20.9 % had borderline and 8.7 % had increased level of cholesterol (Table 2). Borderline level of serum triglyceride was revealed in 13.8 % and high serum level in 14.1 %. In this regard, abnormal increased level of LDL-C and low level of HDL-C were reported in 10.4 and 77.4 % respectively. High cholesterol level was more specified to women than in men, whereas increased level of serum triglyceride was more observed in men. Moreover, women had higher level of LDL-C and lower level of HDL-C compared with men. There was an overall trend of increase in serum cholesterol, triglyceride, and LDL-C levels by increasing age (Table 3).

**Table 2:** The standardized prevalence of abnormal lipid profiles; Cholesterol, Triglyceride, LDL and HDL, Community-Based Cohort Study (KERCADR – 1st Round - *N* = 5558)

	Cholesterol		Triglyceride		LDL-C		HDL-C	
	Borderline	High	Borderline	High	Borderline	High	Borderline	Low
Total	20.9 [19.5,22.2]	8.7 [8.0,9.6]	13.8 [12.6,14.9]	14.1 [13.0,15.2]	21.8 [20.4,23.3]	10.4 [9.6,11.4]	20.3 [18.8,21.9]	77.4 [75.7, 79.0]
Sex								
Male	20.7 [18.8,22.7]	7.8 [6.7,9.1]	15.9 [14.2,17.9]	16.7 [14.9,18.6]	20.6 [18.7,22.7]	9.8 [8.5,11.3]	29.6 [27.0,32.3]	69.6 [66.9,72.2]
Female	21 [19.2,22.9]	9.6 [8.6,10.7]	12 [10.6,13.5]	12.1 [10.8,13.4]	22.9 [20.9,25.0]	11.1 [10.0,12.3]	12.2 [10.7,13.9]	84.2 [82.3,86.0]
Age groups								
15–24	9.5 [7.4,12.2]	1.8 [1.1,3.2]	7.8 [5.9,10.2]	6 [4.4,8.3]	12.5 [10.0,15.4]	2.6 [1.6,4.0]	23 [19.7,26.7]	74.9 [71.1,78.4]
25–34	22.5 [19.6,25.6]	6.2 [4.6,8.2]	12.8 [10.6,15.3]	12.2 [10.0,14.7]	22.1 [19.3,25.2]	8.8 [6.9,11.0]	18.9 [16.3,21.8]	78.9 [75.8,81.7]
35–44	28.8 [25.8,32.0]	12.7 [10.5,15.3]	18.5 [16.0,21.4]	18.9 [16.3,21.8]	29.6 [26.4,32.9]	15.9 [13.4,18.7]	17.8 [15.3,20.6]	79.7 [76.7,82.4]
45–54	33 [30.1,36.1]	18.8 [16.4,21.5]	20.4 [17.9,23.2]	26.7 [23.9,29.6]	33 [30.0,36.2]	20.1 [17.5,22.9]	17 [14.6,19.6]	80.6 [77.8,83.1]
55–64	30.9 [27.8,34.3]	22.2 [19.4,25.2]	22.3 [19.5,25.3]	26.8 [23.8,29.9]	28.6 [25.6,31.9]	23.2 [20.4,26.3]	21.3 [18.5,24.4]	76.4 [73.2,79.3]
65–75	30.2 [25.9,34.8]	20.5 [17.0,24.6]	22.2 [18.5,26.3]	22.7 [19.0,26.9]	29.6 [25.3,34.2]	22.4 [18.7,26.6]	23.5 [19.5,27.9]	72.9 [68.3,77.1]

*TC* total cholesterol, *HDL-C* high density cholesterol, *LDL-C* low density cholesterol, *TG* triglyceride

**Table 3:** The standardized prevalence of dyslipidemia (undiagnosed and diagnosed lipid profiles), Community-Based Cohort Study (KERCADR – 1st Round - *N* = 5558)

Subgroups	N	Normal	Undiagnosed dyslipidemia	Dignosed Dyslipidemia
Overall	5558	18.6 [17.1,20.2]	68.9 [67.2,70.6]	12.5 [11.6,13.3]
Sex				
Men	2501	24.7 [22.2,27.3]	64.6 [61.9,67.2]	10.7 [9.5,12.1]
Women	3057	12.3 [10.6,14.2]	73.4 [71.4,75.4]	14.3 [13.2,15.4]
Age groups				
15–24	806	23.6 [20.2,27.4]	75 [71.2,78.5]	1.4 [0.7,2.6]
25–34	1061	17.4 [14.8,20.3]	75.3 [72.0,78.3]	7.3 [5.6,9.5]
35–44	1037	14.5 [12.2,17.1]	69.3 [66.0,72.5]	16.2 [13.8,18.9]
45–54	1168	11.9 [9.9,14.3]	56.6 [53.3,59.8]	31.5 [28.6,34.6]
55–64	967	12.2 [10.0,14.7]	47.8 [44.2,51.3]	40.1 [36.7,43.6]
65–75	519	15.3 [11.9,19.4]	49.3 [44.4,54.3]	35.4 [30.9,40.1]
Education				
Illiterate	772	20.7 [10.7,36.2]	59.8 [48.1,70.5]	19.5 [10.0,34.6]
Primary to high school	3734	19.1 [17.3,21.0]	68.9 [66.8,70.8]	12.1 [11.1,13.1]
Above high school	1052	16.6 [13.8,19.8]	69.5 [66.0,72.7]	14 [12.1,16.1]
Current cigarette smoker				
No	4937	18.7 [17.1,20.4]	68.4 [66.6,70.2]	12.9 [12.0,13.9]
Yes	621	13.3 [8.9,19.4]	73.3 [65.1,80.1]	13.4 [8.7,20.1]
Opium consumption				
No	4762	18.7 [17.1,20.4]	68.5 [66.7,70.2]	12.8 [11.9,13.8]
Occasional user	459	12.9 [8.5,19.1]	74.9 [69.0,80.0]	12.1 [10.1,14.4]
Depended user	337	28.4 [22.2,35.6]	60.6 [53.0,67.7]	11 [7.9,15.1]
Depression				
No	3394	19.5[17.7,21.6]	68.9 [66.8,70.9]	11.6 [10.6,12.7]
Yes	2164	16.9[14.3,19.9]	70.4 [67.3,73.3]	12.7 [11.4,14.1]
Anxiety				
No	1226	18.8 [15.6,22.5]	71.4 [67.6,74.9]	9.8 [8.2,11.6]
Yes	4332	18.7 [17.0,20.6]	68.2 [66.2,70.1]	13.1[12.1,14.1]
Obesity				
Normal	2533	23.7 [21.6,25.9]	68.1 [65.7,70.3]	8.3 [7.3,9.4]
Overweight	2021	10.2 [8.0,12.9]	73 [69.9,76.0]	16.8 [14.6,19.1]
Obese	1004	8.4 [5.7,12.1]	74.6 [70.2,78.6]	17 [14.2,20.3]
Physical activity				
Low	2456	17.3 [14.8,20.1]	68.7 [65.9,71.5]	14 [12.5,15.5]
Moderate	2612	18.3 [16.2,20.7]	69.6 [67.2,72.0]	12 [10.9,13.3]
High	490	23.2 [18.6,28.6]	68.9 [63.2,74.1]	7.9 [5.7,10.7]
Family History of dyslipidemia				
No	2807	19.5 [17.4,21.7]	70.2 [67.8,72.4]	10.4 [9.3,11.5]
Yes	2751	17.6 [15.2,20.2]	67.9 [65.1,70.5]	14.6 [13.3,16.0]

**Fig. 1:**
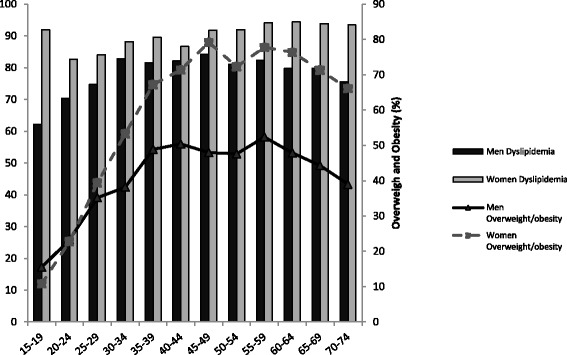
Prevalence of dyslipidemia and overweight/obesity by age group and sex in Kerman, 2011; (Community-Based Cohort Study (KERCADRS – 1st Round - *N* = 5558)

**Fig. 2:**
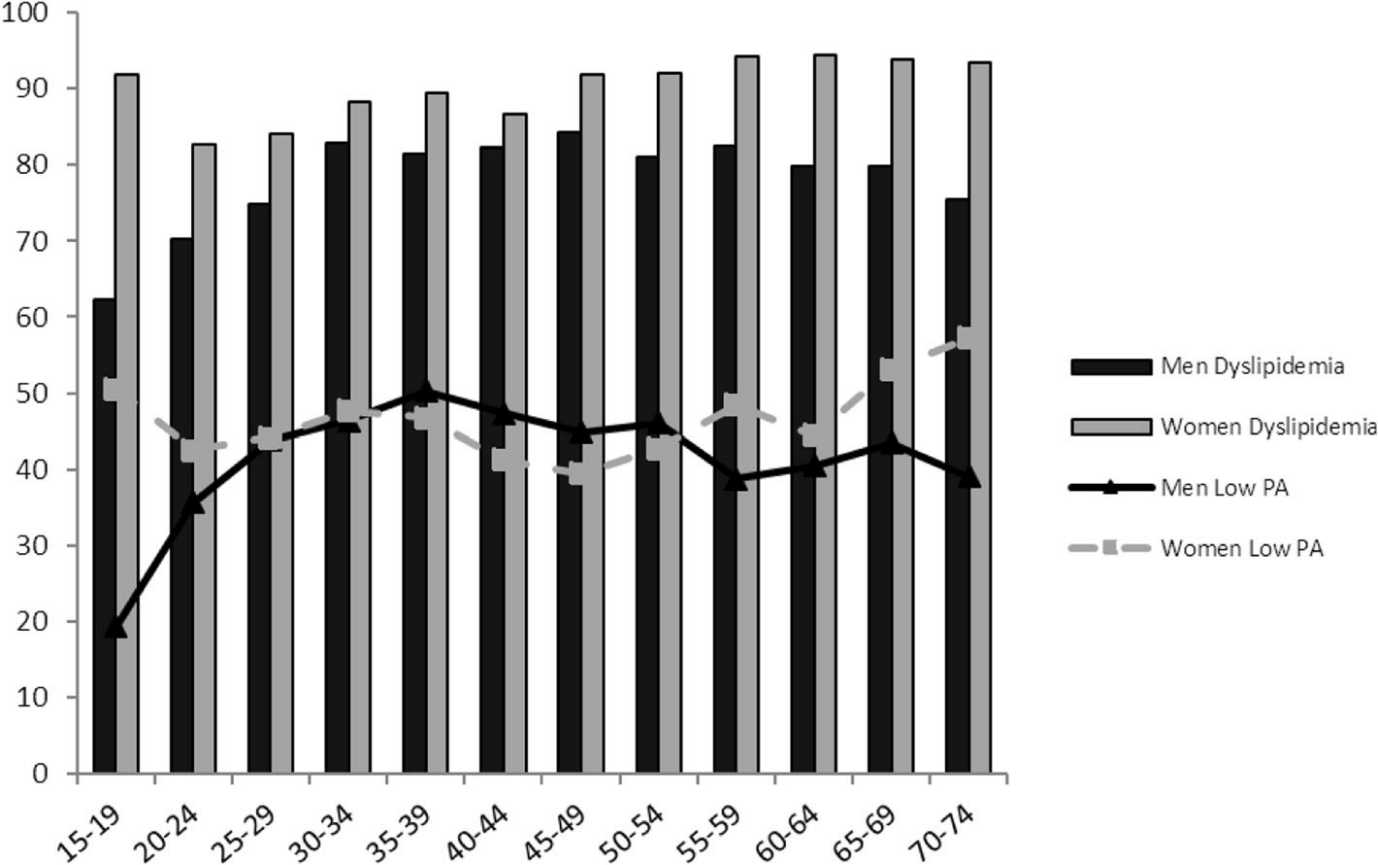
Prevalence of dyslipidemia and Low physical activity (Low PA) by age group and sex in Kerman, 2011; (Community-Based Cohort Study (KERCADRS – 1st Round - *N* = 5558)

The standardized prevalence of dyslipidemia in different gender and age subcategories are shown in Table 3. The overall prevalence of undiagnosed dyslipidemia was 68.9 % and of diagnosed dyslipidemia was 12.5 %. Among subjects with dyslipidemia the prevalence of undiagnosed dyslipidemia was 16.8 % and of diagnosed dyslipidemia was 13.2 % that both types were more prevalent in women. The prevalence of undiagnosed dyslipidemia was significantly higher in educated individuals than in those with lower educational level. Also, smokers had higher prevalence of undiagnosed dyslipidemia than non-smokers. Similarly, opium users had higher prevalence of undiagnosed dyslipidemia compared with non-users. In addition, diagnosed dyslipidemia was more prevalent in depressed than in non-depressed subjects and also in anxious than in non-anxious persons. Meanwhile, overweight and obese cases had higher undiagnosed and diagnosed dyslipidemia compared to those with normal weight. The prevalence of diagnosed dyslipidemia was significantly lower among subjects with high physical activity (7.9 %: 95 % CI, 5.7–10.7 %) than those with low physical activity (14 %: 95 % CI, 12.5–15.5 %). Also, those with positive family history of dyslipidemia suffered more from this lipid abnormality. By adjusting baseline indicators as probable confounders (Table 4), advanced age, anxiety, obesity, and family history of dyslipidemia could effectively predict dyslipidemia in study population. In this context, gender, education level, smoking, opium use and depression states were not found as significant determinants of dyslipidemia. As shown in Fig. 1, the prevalence of dyslipidemia in men increased by fifth decade and then reduced by increase of age, while in women it continued its rising trend by age. The peak age of obesity in men was in the range of 55 to 59 years and in women in the range of 45 to 49 years. Similarly, the peak age of low physical activity in men was in the range of 55 to 59 years and in women in the range of 45 to 49 years (Fig. 2).

**Table 4:** Crude and adjusted odds ratio for different predictors of dyslipidemia, Community-Based Cohort Study (KERCADRS – 1st Round - *N* = (5558)

Subgroups	Crude OR	Adjusted OR
Sex		
Men	---	---
Women	2.4 [2.05,2.9]	2 [1.6,2.5]
Age groups		
15–24	---	---
25–34	1.4 [1.1,1.9]	1.1 [0.8,1.5]
35–44	1.8 [1.3,2.4]	1.1 [0.8,1.5]
45–54	2.2 [1.7,3]	1.3 [1,1.9]
55–64	2.2 [1.6,3]	1.6 [1.1,2.3]
65–75	1.7 [1.2,2.4]	1.2 [0.8,1.8]
Education		
Illiterate	---	---
Primary to high school	0.7 [0.5,1]	1 [0.7,1.5]
Above high school	0.7 [0.5,1]	1 [0.7,1.6]
Current cigarette smoker		
No	---	---
Yes	0.75 [0.59,0.96]	1.1 [0.8,1.5]
Opium consumption		
No	---	---
Occasional user	0.9 [0.6,1.4]	1 [0.7,1.6]
Depended user	0.8 [0.6,1.1]	1.1 [0.7,1.5]
Depression		
No	---	---
Yes	1.4 [1.1,1.6]	1.1 [0.9,1.4]
Anxiety		
No	---	---
Yes	1.2 [1,1.5]	1 [0.8,1.2]
Obesity		
Normal	---	---
Overweight	2.5 [2.1,3.1]	2.3 [1.8,2.9]
Obese	3.3 [2.5,4.4]	2.4 [1.8,3.3]
Physical activity		
High	---	---
Moderate	1.7 [1.3,2.2]	1 [0.7,1.3]
Low	2.2 [1.6,2.8]	1.4 [1,1.9]
Family History of dyslipidemia		
No	---	---
Yes	1.2 [1,1.5]	1 [0.8,1.2]

*OR* odds ratio, Numbers are reported as OR and [95 % Confidence Interval]

## Discussion

The present study aimed to determine prevalence of dyslipidemia based on demographic characteristics as well as medical status of general population and then assesses main correlates of dyslipidemia and its association with other CAD risk factors. In this regard, the overall prevalence of undiagnosed dyslipidemia was 16.8 % and of diagnosed dyslipidemia was 13.2 % that was significantly influenced by advanced age, anxiety, obesity, and family history of dyslipidemia, but not by smoking, education level, opium addiction and depression state. Also, we found a borderline level of cholesterol in 20.9 % and excess level of cholesterol in 8.7 %. These borderline individuals are in risk of being hypercholesterolemic in next few years as this phenomenon was found to increase by advance age. On the other hand the prevalence of undiagnosed dyslipidemia was more than diagnosed dyslipidemia. These statistics show challenge in health education, screening and controlling lipid profile in the population under the study. In agreement with present data a survey in Pakistan revealed that a large proportion of the population had lipid abnormalities and females had significantly greater values of total cholesterol [14]. Study on Jordanian adults showed that almost half of the participants (48.8 %) had elevated serum total cholesterol, 40.7 % had elevated LDL-C, 40.1 % had low HDL-C, and 43.6 % had elevated triglyceride concentrations [15]. In study by Sharifi and colleagues which performed in Zanjan province of Iran, increased total cholesterol (>200 mg/dL) was observed in 35.4 % of the subjects. Except for the hypertriglyceridemia/low HDL-C pattern, which was more common in males, the other abnormal lipid profiles were more common in females [16]. Tabatabaei and her colleagues reviewed and extracted published papers on prevalence of dyslipidemia in Iranian and international journals until September 2011. They found that hypercholesterolemia; high LDL-C and low HDL-C were more prevalent in women, whereas hypertriglyceridemia was more prevalent in men [17]. Also Esteghamati found that the prevalence of hypertriglyceridemia and hypercholesterolemia was 36.4 and 42.9 % respectively in the studied Iranian population and hypercholesterolemia was higher among females and hypertriglyceridemia among males [18]. None of the above studies included considered mental health status as CAD risk factor. The similarity of findings of last three studies with findings of the present study implies that inappropriate effectiveness of our health care system is at the national level.

Another fact was that the peak of prevalence of hyperlipidemia in women was ten years earlier relative to men and this is associated with 10 year earlier peak in low physical activity prevalence (Fig. 2) rendering those susceptible to cardiovascular events. There was more lipid disorder in physically inactive individuals. Also many more obese individuals had undiagnosed dyslipidemia (42 %) and were unaware of it. It is noteworthy that women who were physically inactive and older were more dyslipidemic. The founding that anxiety is a determinant of dyslipidemia along with high prevalence of severe anxiety in our study population (29.1 % in females and 16.7 % in males [11]) is another caution for our health system menageries. These make the conditions more cautious when considering high prevalence of low physical activity of 39.2 % in men and 45.1 % in women [11]. These facts along with more prevalence of undiagnosed dyslipidemia in our study population means that we must reconsider screening program to detect this potentially reversible and major coronary disease risk factor. In this regard as pointed by Ghayour-Mobarhan [19] and others [20] performing appropriate programs for screening and early diagnosing dyslipidemia followed by proper medications resulted in appropriate control of this abnormality. Ghayour-Mobarhan showed that triglyceride, total cholesterol and low density lipoprotein cholesterol were reduced by 27, 20.5 and 22.7 %, respectively and high density lipoprotein cholesterol rose by 8.96 % among 238 hyperlipidemic individuals during one year of attending lipid clinic [19].

Our data point to high prevalence of the other coronary disease risk factors associated with dyslipidemia such as low physical activity (LPA), obesity, anxiety and smoking in dyslipidemic individuals either presented in the current study or reported elsewhere [21, 22]. The positive association between dyslipidemia and LPA observed in the present study (Table 4) has also been shown between LPA with hypertension and opium use [23, 24]. Opium causes reduction of plasma HDL cholesterol and increases the risk of hypertension [25].Therefore our local health program must consider these CAD risk factors in order to decrease CAD burden.

We acknowledge the limitation of our study as a cross-sectional survey. Beside, this study benefited from a relative large sample size, random sampling, and high response rate and included new risk factors such as mental health and opium consumption. For further studies, we recommend monitoring the lipid profile by a longitudinal prospective cohort study. Also it is required to assess the efficacy of local and national intervention programs in managing and control of dyslipidemia.

## Conclusion

In conclusion the results showed overall prevalence of undiagnosed dyslipidemia 16.8 % and of diagnosed dyslipidemia 13.2 % that was significantly influenced by advanced age, anxiety, obesity, and family history of dyslipidemia. Women are more at risk, although the prevalence of dyslipidemia especially borderline dyslipidemia was high in both sexes and needs urgent actions due to mostly young population of the country approaching middle ages in near future.

## Abbreviations

CAD: Coronary artery disease
FPG: Fasting plasma glucose
HbA1c: Hemoglobin A1C
HDL: High-density lipoprotein
KERCADRS: Kerman coronary artery disease risk study
LDL: Low-density llipoprotein
NCEP: National cholestrol education program
TG: Serum triglyceride
